# Medical Legislation

**Published:** 1885-01

**Authors:** 


### Editorial.

### MEDICAL LEGISLATION.

The General Assembly of the State of Georgia, after a session of several weeks, has taken a recess until July next. Beyond the passage of the inevitable tax bill, containing the usual ten dollar professional assessment, it has perfected but few measures of general interest. It has given the customary salute of recognition to our profession and its labors, by raising a standing committee on public health and sanitation.

To this committee was referred a message from the Governor, calling attention to the probable invasion of Asiatic cholera, and to the importance of efficient measures for its prevention, the subsidence of the disease in other countries allaying public apprehension, and the cogitations of the committee resulting in no recommendation of practical value, upon this subject. They did, however, report a bill to facilitate the study of anatomy, by making lawful the procuring of the necessary material for this purpose, without the desecration of cemeteries, or outraging the sentiments or prejudices of the community. It is to be hoped that this bill, at the adjourned session, will become a law. Malpractice by physicians or surgeons subjects them to heavy penalties, ignorance constitutes no legal defense, and the attempt to gain the necessary information in the only method possible is a misdemeanor, often punished by heavy fines or long imprisonment.

The Judiciary Committee has reported and unanimously recommended the passage of a bill to provide for the payment of medical expert witnesses in criminal cases. This bill certainly ought to pass. Physicians are often obliged, at great sacrifice of time and money, to attend and testify in these cases, in counties remote from their place of residence. The law requiring their personal attendance has hitherto made no provision for their compensation. The hardships and injustice involved are so apparent that it is surprising it has not earlier attracted legislative attention.

